# Incident Type 2 Diabetes Risk of Selective Estrogen Receptor Modulators in Female Patients with Breast Cancer

**DOI:** 10.3390/ph14090925

**Published:** 2021-09-14

**Authors:** Yeo-Jin Choi, Keunhyeong Bak, Yoon Yeo, Yongwon Choi, Sooyoung Shin

**Affiliations:** 1 Department of Clinical Pharmacy, Graduate School of Clinical Pharmacy, CHA University, Seongnam 13488, Korea; yjchoi@cha.ac.kr; 2 Department of Clinical Pharmacy, College of Pharmacy, Ajou University, Suwon 16499, Korea; keunhyeong.bak@gmail.com; 3 Department of Industrial and Physical Pharmacy, College of Pharmacy, Purdue University, West Lafayette, IN 47906, USA; yyeo@purdue.edu; 4 Department of Hematology-Oncology, School of Medicine, Ajou University, Suwon 16499, Korea; 5 Research Institute of Pharmaceutical Science and Technology (RIPST), Ajou University, Suwon 16499, Korea

**Keywords:** breast cancer, adjuvant antiestrogen therapy, diabetes

## Abstract

Accumulating evidence indicates a link between diabetes and cancer. Selective estrogen receptor modulators (SERMs) may increase diabetes risk via antiestrogen effects. This study investigated incident diabetes risk of SERM treatment and its effects on metastatic cancer and death prevention in breast cancer survivors. This retrospective cohort study included female patients with early-stage breast cancer, treated with or without SERMs, between 2008 and 2020 in a tertiary care hospital in Korea. Four propensity score-matched comparison pairs were designed: SERM use versus non-use, long-term use (≥1500 days) versus non-use, tamoxifen use versus non-use, and toremifene use versus non-use; then, logistic regression analysis was performed for risk analysis. SERMs in general were not associated with an elevated risk of diabetes; however, when used for ≥1500 days, SERMs—especially toremifene—substantially increased diabetes risk in breast cancer patients (OR 1.63, *p* = 0.048). Meanwhile, long-term SERM treatment was effective at preventing metastatic cancer (OR 0.20, *p* < 0.001) and death (OR 0.13, *p* < 0.001). SERM treatment, albeit generally safe and effective, may increase diabetes risk with its long-term use in women with breast cancer. Further studies are required to verify the association between toremifene treatment and incident diabetes.

## 1. Introduction

A pathophysiological link between diabetes and cancer has been suggested in several studies; diabetic patients are predisposed to cancer and vice versa when compared with their healthy counterparts, mediated by shared risk factors between the two disease states, metabolic alterations, and complications over disease progression, along with adverse effects from prolonged exposure to pharmacologic therapies [1,2,3,4,5,6]. As the second most commonly diagnosed cancer in 2018, accounting for 12.3% of all incident cancer cases, breast cancer affects more than 2 million women worldwide [7,8,9]. However, its mortality rates have declined substantially, likely due to well-established guidelines pertaining to early diagnoses and effective treatments [9,10]. Given that the survival rates are projected to steadily grow, prevention of long-term complications and adverse effects from pharmacological therapies is of crucial importance in enhancing morbidity and mortality of breast cancer survivors. As these patients are likely to receive anticancer treatments repeatedly to prevent cancer progression and metastatic spread, more research is required to have a better insight into the prognostic role of these therapies with respect to metabolic complications, such as incident diabetes risks. 

Among various treatment modalities for breast cancer management—including surgery, radiation, and chemotherapy—endocrine therapy is reserved for patients diagnosed with hormone receptor (HR) (either estrogen receptor (ER) or progesterone receptor (PR)) positive breast cancer [11]. Selective estrogen receptor modulators (SERMs) are classified as antiestrogen agents prescribed for ER-positive breast cancer [9,12]. Unlike aromatase inhibitors, another drug class of endocrine therapy prescribed to reduce estrogen production only in postmenopausal females, tamoxifen—a gold standard SERM agent for ER-positive breast cancer—provides sufficient antitumor activity both in premenopausal and postmenopausal women, thereby improving survival in these patient populations [11]. Previous studies have demonstrated clinical benefits of tamoxifen in female patients in terms of breast cancer treatment and risk reduction [12]. However, management of side effects or complications from long-term exposure to tamoxifen should not be neglected as these patients diagnosed with ER-positive breast cancer are typically treated with tamoxifen as adjuvant therapy for as long as 5 years to 10 years [11]. 

A previous study reported a possible association between type 2 diabetes mellitus (T2DM) and tamoxifen [13]. Evidence is accumulating that estrogen has a role in glucose homeostasis as estrogen receptors are present on pancreatic β cells that produce insulin [14,15]. Hence, estrogen inhibition can lead to insulin resistance and suppress insulin secretion, whereas estrogen replacement in postmenopausal women was shown to decrease diabetes risk in previous studies [14,16]. Moreover, weight gain—an established risk factor for diabetes—was reported in patients receiving tamoxifen [17,18]. These medication-related effects may make breast cancer survivors, already at high risk for metabolic alterations, more vulnerable to new-onset T2DM in the long term. However, despite tamoxifen being the oldest and most-prescribed SERM agent [8,19], not enough post-marketing surveillance studies have been carried out to evaluate its potential impact on metabolic complications in breast cancer survivors. Furthermore, to our knowledge, toremifene—another SERM agent approved for ER-positive breast cancer treatment—has never been investigated with respect to its potential effects on diabetes risks among breast cancer patients. Therefore, the objective of this study is to explore the risk of new-onset T2DM with SERMs use as compared to non-use in women with early-stage breast cancer, with stratified risk analyses per different risk factors, SERM agents, and treatment durations. 

## 2. Results

### 2.1. Characteristics of Study Patients

There were 7638 adult female patients who had hospital encounters associated with breast cancer diagnoses over the 13-year study period. Of those, a total of 167 patients were excluded due to the following reasons at study entry: preexisting T2DM (*n* = 148), metastatic cancer (*n* = 15), and unstable glycemic control (*n* = 4). Resultantly, 7471 patients were screened for study inclusion: 2531 SERM users and 4940 non-users. Baseline characteristics of the initial cohort patients are summarized in Table S1. Then, 1:1 propensity score (PS) matching was performed to control for potential confounding variables at baseline, leading to a total of 5044 patients (2522 in each of the user and non-user comparison cohorts). The baseline characteristics of the PS-matched patients are summarized in Table 1. No significant between-group differences were found post PS matching in terms of patient age, comorbidity, glycemic control, and comedication patterns. The mean ages of the patients were 46.2 ± 9.7 and 49.2 ± 10.8 years in the SERM and control groups, respectively. SERM users were on the therapy for 2.8 ± 1.8 years on average. The mean follow-up period was 7.3 ± 4.4 years among SERM users and 6.5 ± 4.4 years among non-users. 

### 2.2. Study Outcomes: SERM Users Versus Non-Users

The incidence and risk of new-onset T2DM in SERM users as compared to non-users were assessed in the 1:1 PS-matched groups, and the results are summarized in Table 2. The primary outcome event was encountered in 112 (4.4%) patients in SERM users versus 103 (4.1%) patients in non-users. Although the rate of incident T2DM was more frequent in SERM users, the between-group difference in overall risk did not show statistical significance with an odds ratio (OR) of 1.09 (*p* = 0.53). To account for differential effects of SERMs per patient risk factors, the outcome analyses were then stratified by age and Charlson Comorbidity Index (CCI) category as well as comedication patterns with diabetes promoting drugs and oral glucocorticoid over the follow-up period. Overall, no signal of increased risk of T2DM was detected in any of the above strata. There was also no significant difference in glycemic changes observed between groups: the mean change in fasting glucose and glycated hemoglobin (HbA1c) was 4.5 ± 38.9 mg/dL versus 7.2 ± 44.0 mg/dL (*p* = 0.14) and 0.0 ± 0.4% versus 0.1 ± 0.7% (*p* = 0.39) in SERM users and non-users, respectively. Metastatic cancer was newly diagnosed in a comparable number of patients in both groups, while the risk of death was significantly lower in SERM users compared with non-users (OR 0.60, *p* = 0.001). No signal of incident diabetes risk was detected with SERMs use in general as compared to non-use.

### 2.3. T2DM Risk per SERM Agent and Treatment Duration

A series of additional analyses were performed to assess differential effects of SERM agents and treatment durations on the risk of incident T2DM (Table 3). Here, as for the study groups, we only included those patients belonging to each of the SERM agent cohorts (tamoxifen, toremifene) and the distinct treatment duration cohorts (<600, 600 to <1500, and ≥1500 days). The 1:1 PS-matching against control group patients was repeated for individual comparison pairs. After conducting a series of risk analyses, we found that those who received SERM therapy for ≥1500 days were at a significantly higher risk for incident T2DM development than non-users (OR 1.63, *p* = 0.048). When SERMs per individual agent were compared separately against PS-matched control patients, none of the agents were associated with an increased risk of T2DM compared to non-users. However, when treatment duration was considered for each agent, an elevated risk of T2DM was detected with toremifene when used for ≥1500 days (OR 2.75, *p* = 0.007).

### 2.4. T2DM Risk with Long-Term Use of SERM

**Figure 1:**
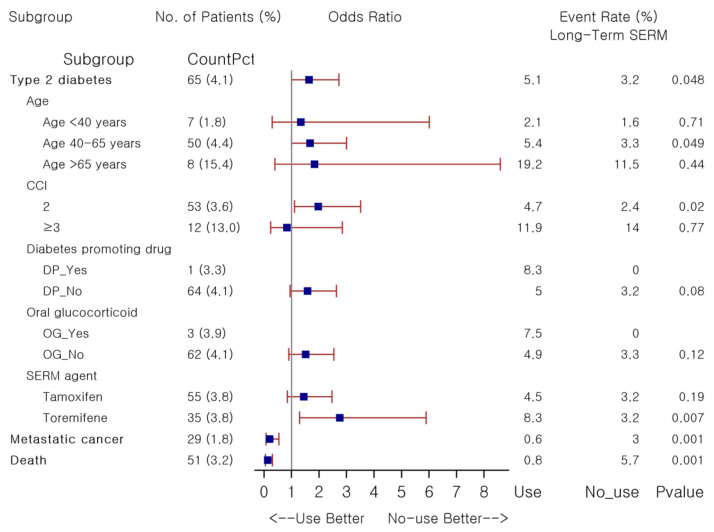
Forest plot for association between study outcomes and long-term use of SERMs in female patients with breast cancer. Abbreviations: SERM, selective estrogen receptor modulator; CCI, Charlson Comorbidity Index; DP, diabetes promoting drug; OG, oral glucocorticoid.

To further investigate risk factors associated with an increased risk of T2DM among those who exposed to SERM for 1500 days or longer, subgroup analyses, stratified by age and CCI category and comedication patterns with diabetes promoting drugs and oral glucocorticoid, were performed between 1:1 PS-matched long-term users versus non-users (Figure 1). Here, we found that incident T2DM risk was substantially elevated in the stratum of 40 years to 65 years (OR 1.67, *p* = 0.049). The incidence of T2DM with long-term use of SERMs was higher in both diabetes promoting drugs exposure and oral glucocorticoid exposure strata, but risk analyses were not performed due to the scarcity of outcome events and limited number of patients belonging to each stratum. When assessed per individual agent, we found elevated risk of T2DM with long-term use of toremifene only (OR 2.75, *p* = 0.007). Glycemic control over the follow-up was comparable between groups as newly diagnosed patients are likely initiated with lifestyle modification measures along with hypoglycemic treatments as needed: the mean change in fasting glucose and HbA1c was 5.4 ± 43.5 versus 4.9 ± 33.3 mg/dL ( *p*= 0.88) and 0.1 ± 0.7 versus 0.3 ± 0.6% (*p* = 0.40) in long-term users and non-users, respectively. The risk of metastatic cancer and death was substantially decreased with long-term use of SERMs as compared with non-use: the OR was 0.20 (*p* < 0.001) and 0.13 (*p* < 0.001), respectively. Here, an increased risk of incident diabetes was assessed when SERM therapy lasted for ≥1500 days as compared to non-use, especially in those aged 40 years to 65 years, with CCI of 2, and who received toremifene. Detailed results are available in Table S2.

### 2.5. T2DM Risk with Tamoxifen

**Figure 2:**
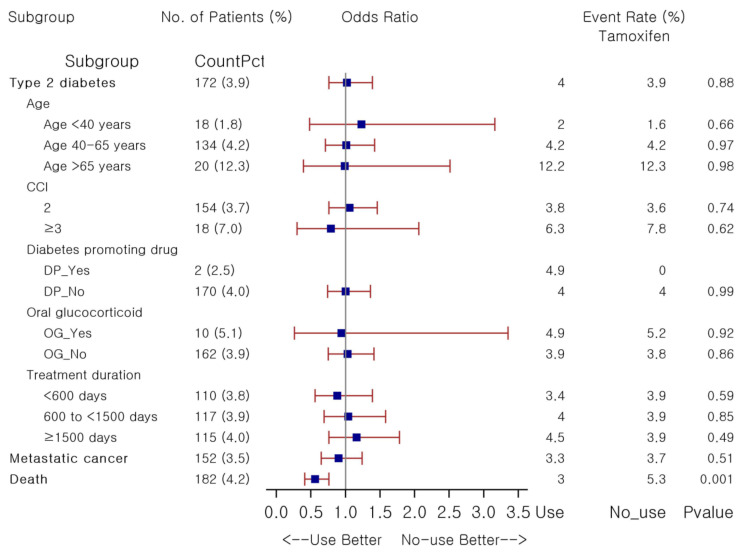
Forest plot for association between study outcomes and tamoxifen in female patients with breast cancer. Abbreviations: CCI, Charlson Comorbidity Index; DP, diabetes promoting; OG, oral glucocorticoid.

To explore potential risk factors associated with tamoxifen, the most frequently prescribed SERM agent, subgroup analyses were conducted using similar stratification methods as above by comparing 1:1 PS-matched tamoxifen users, irrespective of treatment duration, against non-users (Figure 2). No signal of increased risk of T2DM was observed in overall tamoxifen users as compared to non-users, nor in any of the strata per age, CCI, concomitant medications predisposing to diabetes, and treatment duration. Glycemic changes over the follow-up were not significantly different between groups: the mean change in fasting glucose and HbA1c was 4.3 ± 37.4 mg/dL versus 6.9 ± 43.7 mg/dL (*p* = 0.16) and 0.0 ± 0.5% versus 0.1 ± 0.7% (*p* = 0.66) in long-term users and non-users, respectively. Tamoxifen-receiving patients had a significantly lower risk of death than non-users (OR 0.56, *p* < 0.001), although metastatic cancer risk was not significantly different between groups. There was no significant risk of incident diabetes with tamoxifen use as compared to non-use. Detailed results are provided in Table S3.

### 2.6. T2DM Risk with Toremifene

**Figure 3:**
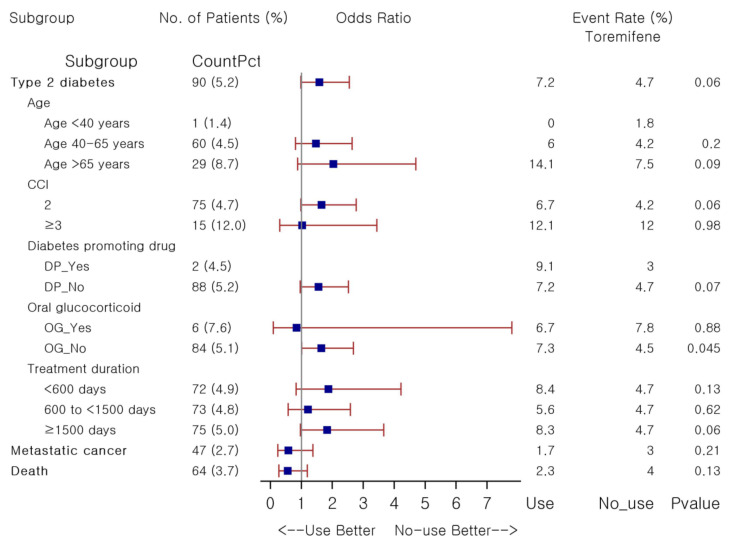
Forest plot for association between study outcomes and toremifene in female patients with breast cancer. Abbreviations: CCI, Charlson Comorbidity Index; DP, diabetes promoting; OG, oral glucocorticoid.

Subgroup analyses were repeated to explore potential risk factors associated with toremifene’s effects on T2DM risk, based on the similar stratification methods by comparing 1:4 PS-matched toremifene users, irrespective of treatment duration, against non-users (Figure 3). Toremifene was not associated with greater risk of T2DM than non-users, nor in most strata, except for the stratum of no oral glucocorticoid exposure, where higher T2DM risk was observed with toremifene use (OR 1.64, *p* = 0.045). Although the rate of new-onset diabetes was 1.8 times higher among long-term toremifene users than in non-users (8.3% versus 4.7%), it did not reach statistical significance (*p* = 0.06) in this PS-matched subgroup analysis. Glycemic changes over the follow-up were not significantly different between groups: the mean change in fasting glucose and HbA1c was 9.6 ± 64.8 mg/dL versus 5.2 ± 38.6 mg/dL (*p* = 0.50) and 0.1 ± 0.3% versus 0.1 ± 0.4% ( *p* = 0.10) in toremifene users and non-users, respectively. No substantial difference was observed in both the risk of metastatic cancer and death between groups. Here, as compared to non-users, a statistically significant risk of incident diabetes was detected in toremifene users who did not receive oral glucocorticoid concurrently. Detailed results are provided in Table S4.

## 3. Discussion

In this study, we assessed the risk of new-onset T2DM along with metastatic cancer and death risks in SERM-treated women with early-stage breast cancer as compared to those never exposed to SERMs. SERMs in general were not associated with elevated risk of T2DM. However, long-term (≥1500 days) use of SERMs—particularly toremifene—substantially elevated T2DM risks in women with breast cancer as compared to non-use. Unlike the findings in a previous study [13], tamoxifen was not associated with increased risk of T2DM in the current study. These findings may have been affected by different characteristics of study patients in the present study relative to the previous study by Lipscombe et al. In their study, only older women with breast cancer, aged 65 years or above, were included [13], whereas 94.2% of study patients in the present study were below or equal to 65 years old. Interestingly, a signal of higher diabetes risk was detected with toremifene, especially with long-term use over 1500 days. Given that toremifene is primarily used in postmenopausal women as opposed to tamoxifen whose use is recommended in both pre- and post-menopausal women with ER/PR positive breast cancer [11], toremifene users tend to be older than tamoxifen users, which may have played a role in differential outcomes per SERM agent. To the best of our knowledge, this is the first study that incorporated both tamoxifen and toremifene—two SERM agents approved to treat breast cancer—in risk analyses stratified by patient factors, high-risk comedication patterns, and treatment duration to evaluate their effects on incident diabetes in female breast cancer survivors.

Meanwhile, beneficial effects were detected with long-term use of SERMs on both metastatic cancer and death prevention in study patients, although the type of breast cancer may also have played a role in these findings. Breast cancers can be characterized as HR positive (either single or double HR positive), human epidermal growth factor receptor 2 (HER2) positive, or triple negative, and the type of cancer determines the anticancer treatment modalities and also predicts tumor sensitivity to pharmacological therapies as well as patient prognosis. Of these, HR positive breast cancer is the major type—accounting for more than 70% of all breast cancers—and considered more benign than dual or triple negative cancers [20]. In the current study, this might have contributed to the better outcomes with SERM users than with non-users in terms of the prevention of both metastatic spread and death in study patients, in that SERM-receiving patients obviously had the HR positive type. Previous studies suggested inferior efficacy of tamoxifen in single HR positive (ER+/PR−) to double HR positive (ER+/PR+) breast cancers [20]. Hence, further factor analysis on the prognosis and mortality associated with SERM treatment is warranted.

Diabetes increases the risk of site-specific cancers, including breast cancer [21,22]. According to previous studies, diabetic females have an elevated risk of breast cancer development and all-cause mortality [23,24]. However, the risk of metabolic complications, especially diabetes, among breast cancer patients has not been fully elucidated despite the steady growth in survival rates [9,10] and amid accumulating evidence on the pathophysiological association between diabetes and breast cancer [1,2,3,4,5,6]. This study demonstrated that long-term SERM treatment of ≥1500 days markedly increased the risk of T2DM (OR 1.63, *p* = 0.048). The incidence of T2DM was relatively higher in those patients concurrently receiving other medications known for diabetes risk, such as oral glucocorticoid and diabetes promoting drugs, implying that SERM treatment may place women with breast cancer, already predisposed to metabolic complications, at higher risk of new-onset diabetes in the long term.

In the PS-matched risk analysis where long-term use of SERMs was compared against non-use, the majority of patients belonged to the age group of 40–65 years (72.4%) and CCI scores of 2 (94.2%), indicating that study patients here were mostly not elderly and had no comorbidity other than breast cancer at baseline. These characteristics likely correlate with the study inclusion criteria: only those with early-stage breast cancer were eligible for study entry. Long-term use of SERMs showed a substantially higher risk of T2DM than non-use, especially among patients aged 40–65 years (OR 1.67, *p* = 0.049) and those with a CCI score of 2 (OR 1.97, *p* = 0.02). Increasing age, usually above 45 years, is a major risk factor for T2DM, and menopause also disturbs glucose homeostasis and insulin sensitivity, further elevating diabetes risks [25]. In the present study, although the incidence of T2DM was more frequent with long-term use of SERMs than with non-use in elderly females (over 65 years), no significant between-group difference in T2DM risks was detected in these older patients. This might have been affected by the limited number of study patients belonging to this older age group, possibly due to the availability of aromatase inhibitors as a secondary option in older women, especially those with elevated thromboembolism risks [11,26]. Thus, caution is advised when assessing SERM-induced T2DM risk in postmenopausal women with breast cancer.

Antiestrogen activity of SERMs increases T2DM risk in breast cancer patients as insulin homeostasis is regulated in an estrogen-dependent manner [13,14,15]. However, despite similar antiestrogen activity, the risk of T2DM was substantially higher with toremifene (OR 2.75, *p* = 0.007) but not with tamoxifen (OR 1.44, *p* = 0.19). Toremifene has a similar drug structure to tamoxifen with the addition of a chloride, but possesses more favorable adverse event profiles than the latter: reduced risks of endometrial cancer and thromboembolism [27,28,29]. Nonetheless, the increased risk of T2DM with toremifene may have been influenced by the inherent disparity in patient populations since toremifene is recommended in postmenopausal women only, whereas tamoxifen can be used in both pre- and post-menopausal women with HR positive breast cancer [11]. Therefore, further post-marketing surveillance study on SERMs with a larger sample size that equally represents both pre- and post-menopausal women is needed to verify agent-specific long-term effects on diabetic complications in women with breast cancer.

It is essential to continuously monitor and assess adverse events associated with SERMs reflecting new safety signals, considering the long treatment duration—5-years in most cases—in breast cancer patients. However, there have been insufficient data pertaining to post-marketing surveillance of both tamoxifen and toremifene with respect to their potential impact on metabolic complications among breast cancer survivors. Prevention of adverse effects and complications from long-term exposure to cancer treatment is crucial in order to improve the morbidity and mortality of these patient populations. This study confirmed that SERMs were effective at preventing metastatic progression of cancer and death, consistent with previous studies [30]. However, a risk versus benefit analysis is warranted in the therapeutic optimization process for individual patients in consideration of their potential risk for diabetes in the long term.

This study has several limitations. First, it was a retrospective cohort study based on patient data from single-center electronic medical records (EMRs). Thus, our findings may not be generally applicable to other clinical settings. Any missing or incorrect documentation of diagnosis, medication use, and laboratory values may have influenced the assessment of baseline comorbidities, comedication patterns, and study outcomes. Second, because of the inherent heterogeneity between SERM users and non-users in clinical settings, breast cancer types could not be balanced via PS matching or multiple logistic regression analysis. Third, due to the small sample size and rarity of outcome events, further studies are required to confirm the association between toremifene and new-onset diabetes risk. Additionally, although raloxifene and bazedoxifene—SERM agents prescribed for osteoporosis treatment or breast cancer prophylaxis in postmenopausal women—were not included as a study drug, its potential effects on incident diabetes development also need to be investigated to have a better understanding in SERMs’ class effects versus agent-specific effects. Despite the limitations, the findings of the current study suggested clinically important risks associated with SERMs use in women with early-stage breast cancer. Further research with a larger sample size representing each SERM agent is warranted to provide risk-based, personalized therapeutic optimization in long-term management of breast cancer.

## 4. Materials and Methods

### 4.1. Study Design and Cohort

This retrospective cohort study included adult women with breast cancer, identified per International Classification of Disease, Tenth Revision (ICD-10) diagnosis code and who received an inpatient and/or outpatient care in a tertiary care hospital in Korea during the patient recruitment period between 1 January 2008 and 30 June 2020. Eligible patients were categorized into two cohorts (SERM users versus non-users), and only those with a follow-up period greater than one year were eligible for study inclusion. SERM agents in this study included tamoxifen and toremifene, but not raloxifene and bazedoxifene because they are not approved to treat breast cancer. Predetermined exclusion criteria were underlying T2DM or metastatic cancer at baseline. We also excluded those patients diagnosed with advanced-stage breast cancer (stages 3 and 4) prior to study entry, identified via surgery history and EMRs reviews. Hence, only those with early-stage breast cancer were screened for study entry. For outcome analysis, study patients were followed up until December 2020. This research was a retrospective study approved by the Institutional Review Board (IRB) and the requirement of informed consent was waived by the Ajou University Hospital IRB (AJIRB-MED-MDB-19-309).

### 4.2. Study Medications and Variables

Breast cancer patients treated with SERMs for more than 90 consecutive days were categorized as SERM users and those not exposed to SERMs as non-users. To assess differential effects of the hormonal therapy, users were further classified into three cohorts in accordance with their treatment duration: <600, 600 to <1500, and ≥1500 days. Prespecified variables included patient demographics, comorbidity status along with CCI, use of other medications, such as aromatase inhibitors, diabetes promoting drugs (thiazide diuretic, beta blocker, angiotensin-converting enzyme inhibitor, angiotensin receptor blocker, statin, and antipsychotic agent), oral glucocorticoid, estrogen, progesterone, and adjuvant chemotherapy. The following comorbid conditions were identified per ICD-10 code: cardiovascular disease, end-stage renal disease, stroke, and venous thrombosis.

### 4.3. Study Outcomes

The primary outcome was the incidence and risk of new-onset T2DM in female patients with breast cancer receiving SERM therapy versus those not exposed to such treatment. The outcome analysis was stratified by age category, and exposure to diabetes promoting drugs or oral glucocorticoid, in order to account for differential effects of SERMs per different patient factors. The secondary outcomes were newly-diagnosed metastatic cancer and all-cause death. These outcomes were identified via hospital visit episodes associated with the respective events over the follow-up period. The index date for each study participant was defined as the first hospital encounter date associated with a breast cancer diagnosis. A series of additional cohort risk analyses were performed to better evaluate differential effects of SERMs per individual agent and treatment duration on the risk of incident T2DM, by repeating PS matching against the non-user control group to design each comparison pair. Patient follow-up began on the index date and continued until the earliest occurrence of any of the following censoring events: study endpoint events, follow-up discontinuation prior to the study end date or 31 December 2020, allowing a follow-up period up to 13 years. To improve the reliability and quality of this retrospective study, when it comes to SERM users, only those cases that occurred at least 180 days after therapy initiation were counted as an eligible event and included in risk analysis.

### 4.4. Statistical Analysis

To balance potential confounding factors between SERM users and non-users, eligible patients were matched in a 1:1 or 1:4 ratio to each of the two comparison groups, based on PS matching per relevant pretreatment variables in terms of age category, comorbidity at study entry, and comedication patterns during the follow-up. The PS matching method was then repeated to identify control group patients for each of the following seven comparison pairs: user group patients belonging to four different treatment duration cohorts (<600, 600 to <1500, and ≥1500 days) versus the corresponding number of non-users, plus two user group patients per SERM agent versus the corresponding number of non-users for each comparison pair, respectively. The multinomial PS for individual patients was estimated by fitting a logistic regression model accounting for the aforementioned baseline characteristics as covariates. The caliper matching method was utilized to improve the quality of matching due to heterogeneity with baseline glycemic control and comorbidity status between cohorts. The multiple logistic regression analysis was then used to further adjust for potential confounders among PS-matched patients. Between-group differences in the risk of each endpoint were then evaluated by computing ORs along with 95% confidence intervals (CIs). The *p*-values were two-sided and considered statistically significant if <0.05. Statistical analyses were performed using SAS 9.4 software (SAS Institute Inc., Cary, NC, USA).

## 5. Conclusions

This study found that SERM treatment in general was not associated with elevated risk of incident T2DM in women with early-stage breast cancer. However, a higher risk of new-onset diabetes was detected in those exposed to SERMs for ≥1500 days, especially to toremifene, as compared to non-users. Although long-term SERM treatment was effective in preventing metastatic cancer and death in breast cancer survivors, risk-based personalized therapeutic approach needs to be considered since long-term exposure to SERMs may worsen a preexisting risk of diabetes in these patients, already predisposed to metabolic complications.

## Figures and Tables

**Table 1:** Baseline characteristics of female breast cancer patients post PS matching (*n* = 5044).

Characteristics	SERM Use (*n* = 2522)	Non-Use (*n* = 2522)	*p*-Value
Age (years), mean ± SD	46.2 ± 9.7	49.2 ± 10.8	
<40	510 (20.2)	509 (20.2)	1.00
40–65	1866 (74.0)	1867 (74.0)	
>65	146 (5.8)	146 (5.8)	
CCI, mean ± SD	2.1 ± 0.3	2.1 ± 0.3	
2, *n* (%)	2361 (93.6)	2369 (93.9)	0.64
≥3, *n* (%)	161 (6.4)	153 (6.1)	
Comorbidity			
Cardiovascular disease	84 (3.3)	87 (3.4)	0.82
ESRD	2 (0.1)	4 (0.2)	0.41
Stroke	9 (0.4)	14 (0.6)	0.30
Venous thrombosis	2 (0.1)	1 (0.0)	0.56
Fasting glucose	100.6 ± 21.4	101.8 ± 21.1	0.06
HbA1c	6.8 ± 1.5	6.5 ± 1.4	0.10
Comedication			
Aromatase inhibitor	22 (0.9)	15 (0.6)	0.25
Diabetes promoting drug	51 (2.0)	50 (2.0)	0.92
Oral glucocorticoid	113 (4.5)	113 (4.5)	1.00
Estrogen	2 (0.1)	0 (0.0)	0.16
Progesterone	24 (1.0)	22 (0.9)	0.77
Adjuvant chemotherapy	106 (4.2)	106 (4.2)	1.00
SERM agent			
Tamoxifen	2176 (86.3)	-	-
Toremifene	346 (13.7)	-	

Notes: Diabetes promoting drugs are thiazide diuretic, beta blocker, angiotensin-converting enzyme inhibitor, angiotensin receptor blocker, statin, and antipsychotic agent. Abbreviations: PS, propensity score; SERM, selective estrogen receptor modulator; SD, standard deviation; CCI, Charlson Comorbidity Index; ESRD, end stage renal disease; HbA1c, glycated hemoglobin.

**Table 2:** PS-matched analysis of clinical outcomes for SERM use versus non-use (*n* = 5044).

Outcomes	SERM Use (*n* = 2522)	Non-Use (*n* = 2522)	OR (95% CI)	*p*-Value
Type 2 diabetes	112 (4.4)	103 (4.1)	1.09 (0.83–1.43)	0.53
Age				
<40	10/510 (2.0)	8/509 (1.6)	1.25 (0.49–3.20)	0.64
40–65	83/1866 (4.4)	80/1867 (4.3)	1.04 (0.76–1.42)	0.81
>65	19/146 (13.0)	15/146 (10.3)	1.31 (0.64–2.68)	0.47
CCI				
2	100/2,361 (4.2)	91/2369 (3.8)	1.11 (0.83–1.48)	0.49
≥3	12/161 (7.5)	12/153 (7.8)	0.95 (0.41–2.18)	0.90
Diabetes promoting drug				
Yes	3/51 (5.9)	0/50 (0.0)	-	-
No	109/2471 (4.4)	103/2472 (4.2)	1.06 (0.81–1.40)	0.67
Oral glucocorticoid				
Yes	6/113 (5.3)	5/113 (4.4)	1.21 (0.36–4.09)	0.76
No	106/2409 (4.4)	98/2409 (4.1)	1.09 (0.82–1.44)	0.57
Metastatic cancer	78 (3.1)	81 (3.2)	0.96 (0.70–1.32)	0.81
Death	74 (2.9)	121 (4.8)	0.60 (0.45–0.81)	0.001

Notes: *p*-values were calculated with Chi-square test (Fisher’s exact test) for categorical variables. Statistically significant *p*-values are highlighted in bold. Diabetes promoting drugs are thiazide diuretic, beta blocker, angiotensin-converting enzyme inhibitor, angiotensin receptor blocker, statin, and antipsychotic agent. Abbreviations: PS, propensity score; SERM, selective estrogen receptor modulator; OR, odds ratio; CCI, Charlson Comorbidity Index.

**Table 3:** PS-matched analysis of incident type 2 diabetes risk per treatment duration and SERM agent.

Outcome	SERM Use	Non-Use	OR (95% CI)	*p*-Value
SERM agent				
Tamoxifen	87/2185 (4.0)	85/2185 (3.9)	1.02 (0.76–1.39)	0.88
Toremifene	25/346 (7.2)	18/346 (5.2)	1.42 (0.76–2.65)	0.27
Treatment duration (days)				
<600	32/808 (4.0)	31/808 (3.8)	1.03 (0.62–1.71)	0.90
600 to <1500	40/934 (4.3)	33/934 (3.5)	1.22 (0.76–1.95)	0.40
≥1500	40/789 (5.1)	25/789 (3.2)	1.63 (1.00–2.72)	**0.048**
Tamoxifen	30/668 (4.5)	25/789 (3.2)	1.44 (0.84–2.47)	0.19
Toremifene	10/121 (8.3)	25/789 (3.2)	2.75 (1.29–5.89)	**0.007**

Notes: *p*-values were calculated with Chi-square test (Fisher’s exact test) for categorical variables. Statistically significant *p*-values are highlighted in bold. Abbreviations: PS, propensity score; SERM, selective estrogen receptor modulator; OR, odds ratio.

## Data Availability

Data is contained within the article and Supplementary Material.

## Supplementary Materials

The following are available online at https://www.mdpi.com/article/10.3390/ph14090925/s1, Table S1: Baseline characteristics of female breast cancer patients prior to PS matching (*n* = 7471); Table S2: PS-matched analysis of clinical outcomes for long-term (≥1500 days) use of SERM versus non-use (*n* = 1578); Table S3: PS-matched analysis of clinical outcomes for tamoxifen use versus non-use (*n* = 4370); Table S4: 1:4 PS-matched analysis of clinical outcomes for toremifene use versus non-use (*n* = 1730).
