# Supplementary material for: Incident Type 2 Diabetes Risk of Selective Estrogen Receptor Modulators in Female Patients with Breast Cancer
Source: Pharmaceuticals (Basel). 2021 Sep 14;14(9):925. doi: 10.3390/ph14090925 (PMC8472249; doi:10.3390/ph14090925)
Supplement: Supplementary file 1 [file pharmaceuticals-14-00925-s001.zip › pharmaceuticals-1358422-supplementary.pdf]

Table S1. Baseline characteristics of female breast cancer patients prior to PS matching (N=7,471).

Characteristics	SERM use (n=2,531)	No-use (n=4,940)	*p*-value
Age (years), mean±SD	46.2±9.7	51.1±11.1	
<40	519 (20.5)	678 (13.7)	**<0.001**
40 to 65	1,866 (73.7)	3,745 (75.8)	
>65	146 (5.8)	517 (10.5)	
CCI, mean±SD	2.1±0.3	2.1±0.3	
2, n (%)	2,370 (93.6)	4,640 (93.9)	0.62
≥3, n (%)	161 (6.4)	300 (6.1)	
Comorbidity			
Cardiovascular disease	84 (3.3)	181 (3.7)	0.45
ESRD	2 (0.1)	4 (0.1)	0.98
Stroke	9 (0.4)	28 (0.6)	0.22
Venous thrombosis	2 (0.1)	2 (0.0)	0.50
Fasting glucose	100.6±21.4	105.1±27.6	**<0.001**
HbA1c	6.8±1.5	6.8±1.5	0.97
Treatment duration (days)			
<600	808 (31.9)	-	-
600 to <1,500	934 (36.9)	-	
≥1,500	789 (31.2)	-	
Comedication			
Aromatase inhibitor	23 (0.9)	22 (0.4)	**0.01**
Diabetes promoting drug	52 (2.1)	76 (1.5)	0.10
Oral glucocorticoid	117 (4.6)	151 (3.1)	**0.001**
Estrogen	3 (0.1)	0 (0.0)	**0.02**
Progesterone	24 (0.9)	28 (0.6)	0.06
Adjuvant chemotherapy	115 (4.5)	126 (2.6)	**<0.001**
SERM agents			
Tamoxifen	2,185 (84.9)	-	**-**
Toremifene	346 (13.4)	-	

Notes: *p*-values were calculated with Chi-square test (Fisher's exact test) for categorical variables. Statistically significant p-values are highlighted in bold.

Diabetes promoting drugs are thiazide diuretic, beta blocker, angiotensin-converting enzyme inhibitor, angiotensin receptor blocker, statin, and antipsychotic agent.

Abbreviations: SERM, selective estrogen receptor modulator; SD, standard deviation; CCI, Charlson comorbidity index; ESRD, end stage renal disease; HbA1c, glycated hemoglobin.

Table S2. PS-matched analysis of clinical outcomes for long-term (≥1500 days) use of SERM versus no use (N=1,578).

Outcomes	Long-term use of SERM (n=789)	No-use (n=789)	OR (95% CI)	*p*-value
Type 2 diabetes	40 (5.1)	25 (3.2)	1.63 (1.00-2.72)	**0.048**
Age				
<40	4/193 (2.1)	3/191 (1.6)	1.33 (0.29-6.01)	0.71
40-65	31/570 (5.4)	19/572 (3.3)	1.67 (1.00-3.00)	**0.049**
>65	5/26 (19.2)	3/26 (11.5)	1.83 (0.39-8.59)	0.44
CCI				
2	35/747 (4.7)	18/739 (2.4)	1.97 (1.10-3.51)	**0.02**
≥3	5/42 (11.9)	7/50 (14.0)	0.83 (0.24-2.84)	0.77
Diabetes promoting drug				
Yes	1/12 (8.3)	0/18 (0.0)	-	-
No	39/777 (5.0)	25/771 (3.2)	1.58 (0.94-2.63)	0.08
Oral glucocorticoid				
Yes	3/40 (7.5)	0/37 (0.0)	-	-
No	37/749 (4.9)	25/752 (3.3)	1.51 (0.90-2.54)	0.12
SERM agent				
Tamoxifen	30/668 (4.5)	25 (3.2)	1.44 (0.84-2.47)	0.19
Toremifene	10/121 (8.3)	25 (3.2)	2.75 (1.29-5.89)	**0.007**
Metastatic cancer	5 (0.6)	24 (3.0)	0.20 (0.07-0.54)	**<0.001**
Death	6 (0.8)	45 (5.7)	0.13 (0.05-0.30)	**<0.001**

Notes: *p*-values were calculated with Chi-square test (Fisher's exact test) for categorical variables. Statistically significant p-values are highlighted in bold.
Diabetes promoting drugs are thiazide diuretic, beta blocker, angiotensin-converting enzyme inhibitor, angiotensin receptor blocker, statin, and antipsychotic agent.
Abbreviations: PS, propensity score; SERM, selective estrogen receptor modulator; OR, odds ratio; CCI, Charlson comorbidity index.

Table S3. PS-matched analysis of clinical outcomes for tamoxifen use versus no use (N=4,370).

Outcomes	Tamoxifen use (n=2,185)	No-use (n=2,185)	OR (95% CI)	*p*-value
Type 2 diabetes	87 (4.0)	85 (3.9)	1.02 (0.76-1.39)	0.88
Age				
<40	10/505 (2.0)	8/497 (1.6)	1.23 (0.48-3.16)	0.66
40-65	67/1,598 (4.2)	67/1,607 (4.2)	1.01 (0.71-1.42)	0.97
>65	10/82 (12.2)	10/81 (12.3)	0.99 (0.39-2.51)	0.98
CCI				
2	79/2,057 (3.8)	75/2,057 (3.6)	1.06 (0.76-1.46)	0.74
≥3	8/128 (6.3)	10/128 (7.8)	0.79 (0.30-2.06)	0.62
Diabetes promoting drug				
Yes	2/41 (4.9)	0/38 (0.0)	-	-
No	85/2,144 (4.0)	85/2,147 (4.0)	1.00 (0.74-1.36)	0.99
Oral glucocorticoid				
Yes	5/102 (4.9)	5/96 (5.2)	0.94 (0.26-3.35)	0.92
No	82/2,083 (3.9)	80/2,089 (3.8)	1.03 (0.75-1.41)	0.86
Treatment duration (days)				
<600	25/725 (3.4)	85/2,185 (3.9)	0.88 (0.56-1.39)	0.59
600 to <1,500	32/792 (4.0)	85/2,185 (3.9)	1.04 (0.69-1.58)	0.85
≥1,500	30/667 (4.5)	85/2,185 (3.9)	1.16 (0.76-1.78)	0.49
Metastatic cancer	72 (3.3)	80 (3.7)	0.90 (0.65-1.24)	0.51
Death	66 (3.0)	116 (5.3)	0.56 (0.41-0.76)	**<0.001**

Notes: *p*-values were calculated with Chi-square test (Fisher's exact test) for categorical variables. Statistically significant p-values are highlighted in bold.

Diabetes promoting drugs are thiazide diuretic, beta blocker, angiotensin-converting enzyme inhibitor, angiotensin receptor blocker, statin, and antipsychotic agent.

Abbreviations: PS, propensity score; OR, odds ratio; CCI, Charlson comorbidity index.

Table S4. 1:4 PS-matched analysis of clinical outcomes for toremifene use versus no use (N=1,730).

Outcomes	Toremifene use (n=346)	No-use (n=1,384)	OR (95% CI)	*p*-value
Type 2 diabetes	25 (7.2)	65 (4.7)	1.58 (0.98-2.55)	0.06
Age				
<40	0/14 (0.0)	1/56 (1.8)	-	-
40-65	16/268 (6.0)	44/1,060 (4.2)	1.47 (0.81-2.64)	0.20
>65	9/64 (14.1)	20/268 (7.5)	2.03 (0.88-4.70)	0.09
CCI				
2	21/313 (6.7)	54/1,292 (4.2)	1.65 (0.98-2.77)	0.06
≥3	4/33 (12.1)	11/92 (12.0)	1.02 (0.30-3.44)	0.98
Diabetes promoting drug				
Yes	1/11 (9.1)	1/33 (3.0)	-	-
No	24/335 (7.2)	64/1,351 (4.7)	1.55 (0.96-2.52)	0.07
Oral glucocorticoid				
Yes	1/15 (6.7)	5/64 (7.8)	0.84 (0.09-7.80)	0.88
No	24/331 (7.3)	60/1,320 (4.5)	1.64 (1.01-2.68)	**0.045**
Treatment duration (days)				
<600	7/83 (8.4)	65 (4.7)	1.87 (0.83-4.22)	0.13
600 to <1,500	8/142 (5.6)	65 (4.7)	1.21 (0.57-2.58)	0.62
≥1,500	10/121 (8.3)	65 (4.7)	1.83 (0.97-3.66)	0.06
Metastatic cancer	6 (1.7)	41 (3.0)	0.58 (0.24-1.37)	0.21
Death	8 (2.3)	56 (4.0)	0.56 (0.27-1.19)	0.13

Notes: *p*-values were calculated with Chi-square test (Fisher's exact test) for categorical variables. Statistically significant p-values are highlighted in bold.

Diabetes promoting drugs are thiazide diuretic, beta blocker, angiotensin-converting enzyme inhibitor, angiotensin receptor blocker, statin, and antipsychotic agent.

Abbreviations: PS, propensity score; OR, odds ratio; CCI, Charlson comorbidity index.
